# Post-transcriptional modifications caused by TDP-43 mutations in mouse and man

**DOI:** 10.1186/2193-1801-4-S1-L52

**Published:** 2015-06-12

**Authors:** Pietro Fratta

**Affiliations:** UCL – Institute of Neurology and National Hospital for Neurology and Neurosurgery, UK

TDP43 is a ubiquitously expressed prevalently nuclear protein involved in RNA splicing, RNA stability and miRNA processing. Amyotrophic lateral sclerosis (ALS), frontotemporal dementia (FTD) and inclusion body myopathy (IBM) are characterized by TDP43 being depleted from the nucleus and accumulating in cytoplasmic inclusions, which are the pathological hallmark of the disease – these diseases are also defined as “TDP43 proteinopathies”. Mutations in TDP43 have been found to be causative of a proportion of ALS cases reinforcing the primary importance of this molecule in disease pathogenesis. The pathogenic mechanism by which TDP43 acts is unclear, and both loss of nuclear function (LOF) and gain of function (GOF) mechanisms have been proposed. We here discuss how we used muscle tissue, which provides high quality RNA of patient disease tissue, to investigate the consequences of TDP43 mislocalization. Further, we characterize two novel mouse TDP43 mutant lines, carrying ENU-induced mutations in order to study the effects of TDP43 mutations expressed at physiological levels in the mammalian central nervous system. One mutation (deltaRNA) strongly reduces the RNA-binding capacity of the protein; the second mutation (C-TERM) is in the glycine-rich C-terminal domain where the majority of human pathogenic mutations are found. Our results underline the importance of studying models with physiological expression levels of TDP43 mutations and shed light on the different effects on RNA metabolism caused by the TDP43 loss and gain of function.

